# Local Legislation is Associated With Regional Transgender Attitudes

**DOI:** 10.1177/01461672231218340

**Published:** 2023-12-28

**Authors:** Eliane Roy, Eric Hehman, Jordan Axt

**Affiliations:** 1 McGill University, Montreal, Quebec, Canada; 2 Project Implicit, Seattle, Washington, United States

**Keywords:** implicit attitudes, IAT, explicit attitudes, transgender, policy

## Abstract

Using a newly developed measure of implicit transgender attitudes, we investigate the association between state-level antitransgender policies and individual-level attitudes about transgender people among residents. In a large sample of U.S. participants (*N* = 211,133), we find that individuals living in states with more discriminatory policies against transgender people (e.g., not allowing changes to one’s gender identity on official identity papers) exhibited more negative implicit and explicit transgender attitudes. This pattern held after controlling for participant race and gender, as well as when looking only at cisgender participants. These findings extend prior work concerning how intergroup biases relate to regional characteristics such as legislation and do so in a novel and consequential context. This research also informs ongoing work concerning the role of policy-making and social norms on the development and expression of intergroup prejudice.

In recent years, the proportion of people publicly identifying as transgender has increased. Studies estimate that the percentage of people identifying as transgender has doubled in the last decade, reaching around 0.52% of the United States population in 2022 (Herman et al., 2022). Transgender people have also become more prominent in the media, but this increased visibility has been linked to greater stereotyping and stigmatization of transgender people (Mocarski et al., 2019). Another potential consequence of this increased visibility is a surge of legal and political responses across states. For instance, 2019 marked the end of a 3-year judicial battle to remove North Carolina’s “bathroom bill,” which prevented transgender people from using restrooms matching their gender identity in public buildings (Drew, 2019). Around the same time, the state of Idaho attempted to enact a law banning transgender women from competing in women’s sports (AP News, 2020). Currently, 19 U.S. states have passed legislation that bars or limits transgender sports participation (Chen, 2022). These laws align with broader work revealing that transgender people face discrimination in many contexts, such as in health care (Winter et al., 2016), employment (James et al., 2016), and housing (Glick et al., 2020).

The rise in people publicly identifying as transgender, as well as the increase in legislation concerning their treatment, has in turn motivated research on attitudes toward transgender people, but this segment of literature remains relatively sparse. Most of the research on transgender attitudes has focused on explicit attitudes, which are comparatively deliberate and self-endorsed, and rely on direct measures of self-report (Greenwald & Banaji, 1995). Multiple studies have shown that people self-report less warmth toward transgender people than lesbians or gay men (Nagoshi et al., 2008; Norton & Herek, 2013). Negative self-reported transgender attitudes have also been found to be more common among political conservatives in the United States (Norton & Herek, 2013) and more religious people (Kanamori et al., 2017).

While self-reported attitudes are certainly informative, these more controlled and endorsed responses may not reflect the full range of evaluations individuals hold toward an attitude object. Implicit attitudes refer to comparatively automatic associations that are less controllable and less aligned with conscious goals (Greenwald & Banaji, 1995). While explicit attitudes are measured using self-reports, implicit attitudes are assessed using indirect measures, in which attitudes are inferred indirectly from behavioral responses. The most prominent method developed to measure such attitudes is the Implicit Association Test (IAT; Greenwald et al., 1998). The logic behind the IAT is that concepts more frequently activated together will elicit stronger associations than concepts less frequently activated together. As such, the IAT measures implicit evaluations via a person’s reaction time while completing a series of association tasks between a target label (e.g., Black-White, Transgender-Cisgender, etc.) and positive or negative attributes (e.g., disgust, joy, rotten, etc.; Greenwald et al., 1998).

Several studies have shown that explicit and implicit attitudes assess distinct but related constructs (e.g., Nosek, 2007). While measures of implicit attitudes are common in many intergroup domains, such as race, religion, or sexual orientation (Greenwald & Lai, 2020), there is relatively little prior work on implicit attitudes toward cisgender and transgender people. The first such study (Wang-Jones et al., 2018) used a version of the IAT with labels of “transsexual men” and “transsexual women” in comparison to “biological men” and “biological women,” finding an overall preference for biological men and women among both gay and straight participants. However, considering that category labels have a significant influence on measures of implicit attitudes (Govan & Williams, 2004), the use of category labels related to one’s genitals in Wang-Jones et al. (2018)—instead of labels related to gender identity (e.g., “transgender women” vs “cisgender women”)—might detract from the measure’s ability to assess implicit transgender attitudes. Relatedly, past research has found that segregating groups into subgroups can yield results that are unrepresentative of the attitudes toward the group as a whole (Fiske et al., 2002; Sesko & Biernat, 2010), further suggesting that the measure used by Wang-Jones et al. (2018) may not capture attitudes toward transgender people in general.

The largest study to date on implicit transgender attitudes involved the development and validation of an IAT measuring attitudes toward transgender versus cisgender people specifically (Axt et al., 2020). Here, the researchers tested two IATs: one using images of famous transgender people (e.g., Chaz Bono, Laverne Cox) or cisgender people (e.g., Jon Favreau, Meagan Good) and another using text stimuli such as “transgender” and “cisgender.” While both IATs found reliable evidence of more positive associations toward cisgender people relative to transgender people, the image IAT often showed slightly greater internal reliability and stronger predictive validity on outcomes like explicit transgender attitudes, policy advocacy, and self-reported transphobia. Subsequent studies found that the image-based transgender IAT also predicted related outcomes like interest in romantic relationships with transgender individuals, prior contact with transgender people, and gender essentialism (Axt et al., 2020). Finally, the measure demonstrated known groups validity in showing significant differences in performance between transgender and cisgender participants (*d* = .86), which aligns with prior IAT work in other intergroup contexts (e.g., Jost et al., 2004; Westgate et al., 2015). A reliable and valid measure of implicit transgender attitudes then allows researchers to investigate related issues, such as the broader context in which these attitudes develop.

## Regional Estimates of Intergroup Bias

In the first two decades of research on implicit attitudes, the default interpretation of performance on indirect measures like the IAT was as an individual difference. According to this perspective, performance on measures like the IAT assesses a construct that is specific to the individual (Greenwald & Banaji, 1995) and could thus be used as a measure of individual traits. Although it may ultimately be impossible to disentangle the degree to which individuals’ implicit associations are impacted by their own personal beliefs versus their cultural environment (Gawronski et al., 2008), performance on implicit measures was still mostly interpreted as reflecting consequential information about the individual participant (Kurdi & Banaji, 2017; Rae & Greenwald, 2017).

However, in recent years, this individual differences interpretation has been re-examined on both empirical and theoretical grounds. For example, measures of implicit attitudes suffer from low temporal stability at the individual level; in one study (Gawronski et al., 2017) an IAT assessing implicit racial attitudes had only a moderate correlation when taken 2 months apart (*r* = .44) while self-reported racial preference demonstrated much stronger temporal stability (*r* = .88). Yet, despite instability at the individual level, the group-level performance on the IAT was surprisingly similar, as the test means fell within two percentage points over the 2 months period (Time 1: *M* = 0.42; Time 2: *M* = 0.46).

To explain this and other instances of stability among individuals versus groups over time, Payne et al. (2017) have proposed the “bias of crowds” model, which posits that measures of implicit biases reflect the accessibility of biases in a specific context and that this accessibility varies across situations, rather than across individual minds. As a result, the researchers propose that measures of implicit biases should be thought of more as measures of *situations* rather than persons. For empirical work supporting this argument, Vuletich and Payne (2019) reanalysed the data from Lai et al. (2016) regarding various interventions to reduce implicit racial bias, as measured by an IAT. The study pooled participants from a number of college campuses, and found that though several interventions could reduce bias immediately, these effects were short-lived. But in their re-analysis, Vuletich and Payne (2019) found that while individual IAT scores mostly randomly fluctuated for the days following the interventions, the campus-level means returned to their preintervention levels. These results provide support for the idea that implicit attitudes are more the stable properties of environments, relative to individuals.

The “bias of crowds” model then argues that implicit biases could be more representative of the environment characteristics as opposed to individual characteristics. According to Payne et al. (2017), in a given area, some situations will only be influential to certain individuals, while other situations will equally affect all individuals in this region. Nevertheless, when aggregated into a sample, the average level of bias among participants in a shared area should reflect the most widely shared situation in this area, be it a city, state, or country. Consistent with this idea, Hehman et al. (2019) demonstrated that race IAT results aggregated for larger regions showed much greater retest reliability than for smaller regions, such that state-level (*M_r_* = .693) > core-based statistical area-level (*M_r_* = .275) > county-level (*M_r_* = .025).

The original “bias of crowds” model pertains to only implicit attitudes, rather than explicit (i.e., self-reported) attitudes, but aggregating to the level of a large geographic area has often produced strong correlations between the two measures. A recent meta-analysis found an average estimate of *r* =.66 between state-level estimates of implicit and explicit attitudes, with some types of implicit and explicit biases correlating as high as *r* = .94 (Calanchini et al., 2022). Moreover, several recent studies have found similar results when using either implicit or explicit regional aggregates of intergroup bias to predict meaningful outcomes. For example, differences in scholastic disciplinary actions (e.g., suspension and expulsion) between Black and White students were related to county-level measures of implicit and explicit racial bias (Riddle & Sinclair, 2019), meaning that counties where participants showed the greatest amount of negative associations toward Black versus White people on an IAT (or in self-reported prejudice) were also more likely to have greater racial disparities in suspensions of Black versus White students. In a related study, counties with higher levels of anti-Black attitudes, either assessed through an IAT or self-report, showed greater disparities in police traffic stops, specifically such that Black drivers were stopped at higher rates relative to their population in the county (Stelter et al., 2022). Based on these results, this work considers both indirect and direct measures of attitudes as viable measures of prejudice that may be associated with factors that vary regionally.

The prejudices of people living in different regions have also been used to estimate the impact of new and existing policies. Since such policies may vary in implementation across different regions (counties, states, etc.), it can be informative to investigate how policymaking, an inherently *regional* factor, is associated with biases among people living in those regions. One prominent example of this approach used a quasi-experimental design to examine how variability among regions in the legalization of same-sex marriage was associated with subsequent changes in implicit and explicit antigay attitudes in both the United States (Ofosu et al., 2019) and in Europe (Aksoy et al., 2020). Results found that both implicit and explicit attitudes toward gay people became more positive before same-sex legalization but did so at a much faster rate following the legislation’s passing. This work demonstrates that while constituents’ attitudes can influence policymaking, policy changes on a larger, and regional scale may also influence the attitudes of an area’s constituents (Tankard & Paluck, 2016).

## The Present Work

It has become an increasingly pressing issue to understand the causes and consequences of discrimination based on trans-identity. To further this effort, the present research is the first to investigate implicit and explicit transgender attitudes as they may vary regionally. More specifically, we explore how variance in transgender-related policies across U.S. states is related to individuals’ antitransgender prejudices. In addition to further validating the novel transgender IAT, our analysis extends research into intergroup biases varying due to regional factors, as the prior work in this area has focused on only a handful of domains, such as race, sexual orientation, religion, skin-tone, weight, and disability (e.g., Calanchini et al., 2022; Hehman et al., 2021; Johnson & Chopik, 2019).

We anticipate that attitudes toward transgender people should be associated with the characteristics of existing policies that dictate the treatment of transgender people. Specifically, we explore whether the characteristics of policies concerning treatment of transgender people in an area is related to local implicit and explicit transgender biases. Following prior work exploring regional predictors of intergroup biases (e.g., Chin et al., 2020; Devos et al., 2021; Lopez et al., 2022), we used a multilevel analysis that nested individual participants within our geographical unit of interest (i.e., states). We then explored the relationship between state-level legislation and individuals’ implicit or explicit transgender attitudes. The findings from this correlational analysis would align with results from several studies suggesting that policies can favorably or unfavorably affect the perception of societal norms (Eisner et al., 2020; Ofosu et al., 2019), though the present work cannot make claims about the causal role that changes in policy may have on individual prejudices.

## Methods

### Participants

A total of 539,096 participants completed the transgender IAT at Project Implicit (https://implicit.harvard.edu) between April 2, 2020, and July 1, 2022. Since this study primarily focuses on regional comparisons, only participants who provided U.S. state information were retained for analysis (65% of participants who provided location data). Following the IAT *D* scoring algorithm, data from participants who had reaction times faster than 300 ms on more than 10% of the trials were removed from analysis (Greenwald et al., 2003; Nosek, 2007). We performed analyses on the full sample of U.S. participants with attentive IAT performance (*N* = 211,133), as well as the subset of participants who identified as cisgender (*N* = 193,239). The full sample was 72.1% White, 70.2% female and 33.6 years old (*SD* = 13.86) on average. When not restricting by gender identity, the minimum sample per state was 343 (median *n* = 2765).

### Measures

#### Implicit Transgender Attitudes

Implicit attitudes were assessed using the Transgender IAT developed by Axt et al. (2020). During this seven-block IAT, participants were presented with either good words (e.g., “Nice, “Pleasure,” etc.) or bad words (e.g., “Nasty,” “Hurt,” etc.) as attributes. The stimuli consisted of eight images of celebrities (four cisgender, four transgender). Pairs of cisgender and transgender celebrities were matched on race and were of approximately the same age and popularity (estimated using Google search returns). Participants were first shown short descriptions of each celebrity and performed a 24-trial training block where they had to correctly classify the transgender (or cisgender) celebrity image, and images were explicitly labeled as cisgender or transgender. As such, we tested for differences between attitudes toward transgender and cisgender people, without further dividing into subgroups (i.e., not measuring attitudes toward transgender men and transgender women separately). These labels were removed for the IAT, which followed the initial training block. The seven-block IAT was administered according to the design outlined by Nosek et al. (2007). Scores were calculated using the *D* algorithm (Greenwald et al., 2003), such that more positive scores indicated more positive implicit associations toward cisgender versus transgender people.

#### Explicit Transgender Attitudes

Participants completed five items concerning explicit attitudes toward cisgender versus transgender people: one relative preference item, two thermometer items, and two liking indicators. For the relative preference item, participants used a −3 (“I strongly prefer transgender people to cisgender people”) to +3 (“I strongly prefer cisgender people over transgender people”) response scale. For the thermometer items, participants rated how warm or cold they felt toward transgender people and then cisgender people (0 = *Extremely cold*, 10 = *Extremely warm*). A difference score was calculated such that positive scores indicated more warmth for cisgender people over transgender people. The two liking items used a slider response scale, where participants reported how negative or positive they felt toward cisgender or transgender people (1 = *Strongly negative*, 100 = *Strongly positive*). Another difference score was computed for the liking items, with higher values indicating a higher relative positivity toward cisgender people over transgender people. Values from the explicit preference scale, difference score of warmth, and difference score of liking were standardized and then averaged together to represent aggregate explicit transgender attitude scores (average *r* = .70; Axt et al., 2020).

#### Transgender Laws

Inclusivity of state-level laws was operationalized as a “policy tally” of gender identity–related laws, available through a 2020 online report from the Movement Advancement Project (2020). Since 2006, this organization has reported data on treatment of LGBTQ+ communities in the United States. These reports have been cited more than 200 times in papers across multiple fields (e.g., Bakko & Kattari, 2021; Lennon-Dearing & Delavega, 2015; Ross & Grift, 2020). The gender identity policy tally used in this research is a count of laws and policies that contribute to equality for gender nonconforming individuals (Movement Advancement Project, 2020). The policy scores include an evaluation of the policies—or lack thereof—that are currently in place in each state. These cover topics such as relationship and parental recognition, nondiscrimination, religious exemptions, transgender youth, health care, criminal justice, and procedures for updating identity documents.

The MAP policy tally scores policies such that each law that either protects against discrimination or expands rights toward transgender people counts as a single, positive point. Positive laws can then be any law that is protecting transgender people’s rights or at least equating them to those of cisgender people. A positive law can also be one that condemns discrimination on the basis of transgender identity. For instance, a state passing a law prohibiting employment discrimination based on gender identity would receive 1 point. Similarly, a state’s policy tally is reduced by a point if the state has a law that harms or deliberately targets LGBT people. For example, a state allowing health care workers to refuse treatment to transgender clients would be deducted 1 point (Movement Advancement Project, 2020). In some cases, fractions of a point are awarded (or deducted), such as for positive or negative local laws that do not cover the entire state population or for laws that only cover a portion of the possible areas. While the scores are updated frequently on the Movement Advancement Project website as new legislation is approved, we used values from when we finished data collection (July 2022).^1^

If we expect trans attitudes to be related to local legislation, an important precondition is that states have substantial variability on policy tally in the first place. Meeting this assumption, states did indeed vary widely on the policy tally measure. For instance, while California and Connecticut had the highest scores of 20.75, Tennessee occupied the lowest position with a score of −5.75. In all, the average policy value was 10.63 (median = 13.5, *SD* = 9.28).

#### Demographics

Participants completed a 13-item demographic questionnaire, of which we only analyzed data relating to country of residence, sex assigned at birth, gender identity, race and political orientation. We used participant’s birth year and month and calculated the age based on the year and month of study session completion. Participants reported their race based on the following labels: “American Indian/Alaska Native,” “East Asian,” “South Asian,” “Native Hawaiian or other Pacific Islander,” “Black or African American,” “White,” “Other or Unknown,” and “Multiracial.”

#### Gender Identity

As part of the demographics questionnaire, participants reported their sex at birth and their current gender identity as part of the demographics questionnaire. For sex at birth, participants were given the options “male” or “female.” For gender identity, participants were asked to select all that apply, between “male,” “female,” “trans male/trans man,” “trans female/trans woman,” “genderqueer/gender nonconforming,” and “different identity.” Following the procedure outlined by Axt et al. (2020), we classified participants as cisgender if their sex at birth matched their current gender identity (91.52%; see Tate et al. (2013) a similar approach in classifying cisgender versus transgender participants). Participants were classified as transgender (1.92%) if they either (a) selected the “trans male/trans man” or “trans female/trans woman” options when reporting gender identity or (b) reported a gender identity that differed from sex assigned at birth (excluding those who selected “genderqueer/gender nonconforming” or “different identity”).

#### Individual-Level Conservatism

Many studies have highlighted the relationship between political ideology and intergroup attitudes, where findings generally show that conservatives hold more favorable implicit and explicit attitudes toward higher-status groups (and less-favorable attitudes toward lower-status groups) than liberals (Jost et al., 2004; Nosek et al., 2007). In the context of LGBTQ+ people specifically, similar evidence points to a positive relationship between conservatism and implicit bias against gay people, at least at the individual level (Jost et al., 2004). Follow-up analyses then sought to control for conservatism, both at the individual level and state level. Participants’ own conservatism was measured within the demographics questionnaire using a 7-point political ideology self-report scale ranging from 1 (“strongly conservative”) to 7 (“strongly liberal”). Importantly, this variable was rescored for interpretability, such that higher values instead indicated “more conservative.”

#### State-Level Conservatism

We obtained the total vote count for the Republican presidential candidate in each state and the overall total vote count in each state from MIT Election Data and Science Lab (2017). State-level conservatism was operationalized as the percentage of vote toward the Republican candidate in the 2020 Presidential Election in the United States.

### Analytic Approach

**Figure 1.**
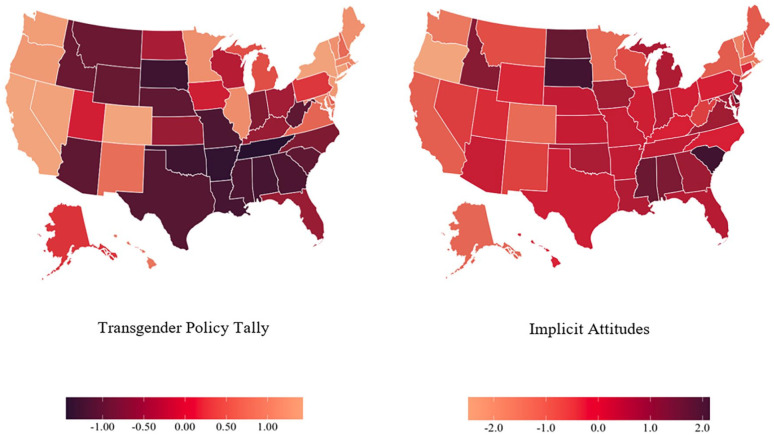
Geographical Representation of State-Level Transgender Policy Tally and Implicit Attitudes. *Note.* Darker values used to represent states with more discriminatory policies and/or with greater levels of implicit biases. Lower values mean more discriminatory laws toward transgender people for Transgender Policy Tally. Higher values mean more negative attitudes toward transgender people for Implicit Attitudes.

Our primary analysis used a multilevel approach to analyze the relationship between state-level transgender policy score (our predictor) with either implicit or explicit transgender attitudes. Participants were geolocated by postal code and then nested in the state where they completed the IAT. We analyzed this relationship for all participants as well as cisgender participants only. See Figure 1 for a map (using the full sample) showing each state’s average transgender IAT *D* score, as well as a map displaying each state’s score on the policy measure (where lower values indicate more discriminatory laws toward transgender people).

In separate models, we regressed implicit and explicit biases on state policy tally. Participants (level 1: *n* = 211,133) were nested within states (level 2: *k* = 50), the level at which the state policy tallies varied. State policy tally (and all other level 2 variables) were grand-mean centered, and models included a random intercept by state. Analysis was done in R using *lme4* (Bates et al., 2015), *r2mlm* (Shaw et al., 2020), and *psych* (Revelle, 2022). *P* values and confidence intervals were derived from Satterthwaite approximations in the *lmerTest* package (Kuznetsova et al., 2017). For each primary analyses, we report the total proportion of variance explained by each model (*R*2), and the proportion of this total variance explained that is attributable to variance between states (*R*2b).

For the analyses controlling for demographics, age was grand-mean centered, race was contrast coded (White participants as −1, and non-White participants as 1) and gender was also contrast coded (“Female” as −1, and “Male” as 1). For analyses exploring the role of conservatism, both individual-level and state-level conservatism were grand-mean centered.

### Procedures

All participants completed the transgender IAT, explicit attitude measure, and demographics questionnaire in a random order. Participants also completed one of three other self-report scales, though these data were not relevant to the primary analyses. The transgender policy tally data were obtained from Movement Advancement Project (2020). All materials for the study are available at https://osf.io/pyr3q/?view_only=e27392f4e5a14928bd56f4bd002a6f75, and data and analysis scripts can be accessed at https://osf.io/pyr3q/?view_only=e27392f4e5a14928bd56f4bd002a6f75.

## Results

### Descriptive Analyses

First, we explored the correlation between average implicit and explicit transgender attitudes across states (*k* = 50). At the state-level, we found a positive and significant relationship between average implicit and explicit attitudes, *r* = .79, 95%, confidence interval [CI]: [.64, .89], *p* < .001, which is consistent with similar analyses in other domains (Hehman et al., 2019).

We also report implicit-explicit correlations at the county-level (*n* = 195; limited to counties with at least 50 observations) to compare with prior work in other domains. At the county-level, average implicit and explicit attitudes correlated strongly, *r* = .60, 95% CI [.45, .73], *p* < .001. County-level results appear to be mostly consistent with previous meta-analytic evidence for the correlation between implicit and explicit attitudes (Calanchini et al., 2022).

As outlined above, our primary analyses use individual-level measures of implicit and explicit transgender attitudes. At the individual-level, the implicit-explicit correlation was positive and medium-to-large (*r* = .35), which is similar to the correlations found in previous work using this transgender IAT (*r* = .30; Axt et al., 2020). Yet because implicit and explicit attitudes were measured with quite different approaches, we consider any similarity in patterns of results when using each of these measures (and similarly, with the full or cisgender-only sample) to be converging evidence. We interpret consistency across these different model specifications to suggest that any conclusion is robust to these researcher decisions.

### Primary Analyses

For all models, we first ran a null (unconstrained) model to partition the variance of implicit or explicit attitudes between states, individuals, and the residual or error variance (Raudenbush & Bryk, 2002). Across the different models, the ICC (intraclass correlation) revealed that between 0.1% and 0.3% of the variance in attitudes toward transgender people was attributable to differences between the states in which participants lived (see Tables 1 and 2 for full reporting). As anticipated, these results reveal that individual transgender attitudes vary much more within than between states.

**Table 1.** Predicting Transgender Implicit Attitudes From Transgender State Policy Tally for the Full and Cisgender-Only Samples.

Parameter or estimate	Implicit attitudes			
	Full sample		Cis-only sample	
	Null model	Model with predictor	Null model	Model with predictor
Fixed effects				
Intercept	.114 (.00)***	.114 (.00)***	.144 (.00)***	.144 (.00)***
State policy tally		−.002 (.00)***		−.001 (.00)***
Variance components				
State intercept variance	.001 (.02)	.0003 (.02)	.0004 (.02)	.0002 (.02)
Residual variance	.199 (.45)	.199 (.45)	.190 (.44)	.190 (.44)
Estimated between state *R*^2^		.43		.42
ICC	.003		.002	

*Note.* ICC = intraclass correlation.

** *p* < .01. ***p < .001.

**Table 2:** Predicting Transgender Explicit Attitudes From Transgender State Policy Tally for Full and Cisgender-Only Samples

Parameter or estimate	Explicit attitudes			
	Full sample		Cis-only sample	
	Null model	Model with predictor	Null model	Model with predictor
Fixed effects				
Intercept	.043 (.02)**	.04 (.01)***	.155 (.01)***	.154 (.01)***
State policy tally		−.009 (.00)***		−.008 (.00)***
Variance components				
State intercept variance	.012 (.11)	.004 (.07)	.01 (.10)	.004 (.06)
Residual variance	.992 (.996)	.992 (.996)	.824 (.91)	.824 (.91)
Estimated between state *R*^2^		.61		.62
ICC	.012		.012	

*Note.* ICC = intraclass correlation.

** *p* < .01. ***p < .001.

This result is consistent with previous work examining implicit or explicit intergroup attitudes clustered by regional units (e.g., metropolitan areas, ICC = 0.019, Devos et al., 2021; counties, ICC = 0.019, Yogeeswaran et al., 2023; states, ICC = 0.014, Lopez et al., 2022; ICC = 0.006, Chin et al., 2020). Yet as this other work has revealed, practically consequential variation in attitudes resulting from regional factors can still emerge, despite large within-state heterogeneity. As per recommendations from Luke (2004), we moved forward with this model given our interest in a relationship between a state-level predictor (state transgender policy tally) and an individual-level outcome (attitudes toward transgender people).

**Figure 2.**
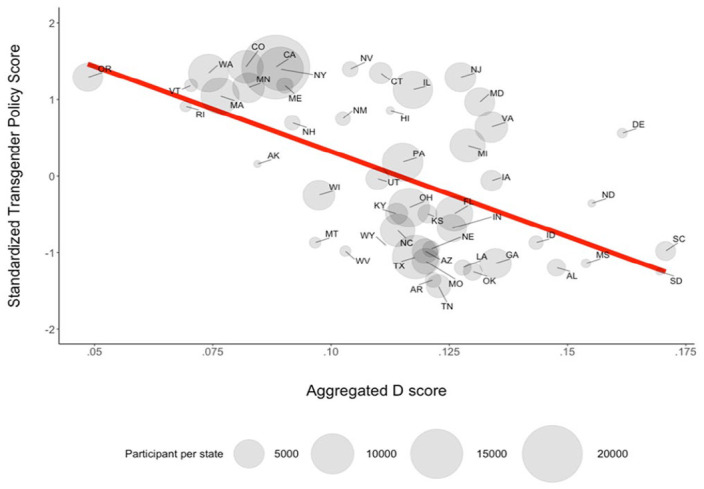
Negative Relationship of State-Level Transgender Policy Tally and Implicit Attitudes.

Next, we turned to the primary models on which we used state-level policy scores to predict residents’ biases. For the complete sample, results showed that people living in states with more discriminatory policies had higher levels of implicit bias, *b* = −0.0016, 95% CI [−0.0022, −0.001], *t*(43.25) = −5.289, *R*2= .003, *R*2b = .43, *p* < .001. See Figure 2 for plot showing the relationship between each state’s aggregate IAT *D* score and score on the policy measure. The same negative relationship was found for explicit attitudes, *b* = −0.009, 95% CI [−0.011, −0.007], *t*(41.23) = −8.28, R2 = .011, R2b = .61, *p* < .001, such that people living in states with more discriminatory policies had higher levels of explicit bias.

The same pattern, and very similar parameter estimates, were found for the cisgender only sample, where greater bias against transgender people on both implicit, *b* = −0.0014, 95% CI [−0.002, −0.001], *t*(42.99) = −4.97, *R*2 = .002, *R*2b = .42, *p* < .001, and explicit attitudes, *b* = −0.008, 95% CI [−0.010, −0.006], *t*(40.67) = −8.33, R2= .011, R2b= .62, *p* < .001, was related to more antitransgender policies. Converging evidence across both implicitly and explicitly measured biases, and the full versus cis-gender only sample, indicate that these measurement and analytic decisions do not impact the overall relationship between state-level policies and antitrans attitudes.

#### Controlling for Demographics

We performed robustness checks of this relationship in subsequent analyses by introducing demographic controls. First, when controlling for age, transgender policy characteristics were still significantly associated with implicit, *b* = −0.002, 95% CI [−0.003, −0.002], *t*(43.45) = −6.38, *R*^2^ = .04, *p* < .001, and explicit transgender attitudes, *b* = −0.01, 95% CI [−0.012, −0.007], *t*(41.91) = −8.45, *R*^2^ = .04, *p* < .001. Results were consistent when controlling for race, implicit: *b* = −0.002, 95% CI [−0.002, −0.001], *t*(44.23) = −5.61, *R*^2^ = .002, *p* < .001; explicit: *b* = −0.009, 95% CI [−0.011, −0.007], *t*(41.16) = −8.17, *R*^2^ = .007, *p* < .001, and gender, implicit: *b* = −0.0016, 95% CI [−0.002, −0.001], *t*(43.3) = −5.32, *R*^2^ = .001, *p* < .001; explicit: *b* = −0.009, 95% CI [−0.011, −0.007], *t*(41.19) = −8.27, *R*^2^ = .007, *p* < .001. In the online supplement, we also provide results of analyses showing that transgender policy characteristics were still a significant predictor of implicit and explicit attitudes when controlling for age, race, and gender in the same model. Ultimately, the relationship between transgender laws and attitudes were robust to these demographic controls.

#### The Role of Conservatism

Finally, we anticipated that there would be strong associations between (a) overall political conservatism of the state, (b) the conservatism of individual participants within the state, and (c) the extent to which a state adopted antitransgender laws, which are broadly considered to be driven by conservative legislatures (Astor, 2023). Indeed, a series of analyses (see online supplement for full reporting) showed a reliable association between state-level conservatism with both conservatism of individual participants (*r* = .86) as well as the measure of antitransgender policies (*r* = −.87), meaning that more conservative states had either fewer laws that expanded transgender rights or more laws that were considered discriminatory to transgender people. Individual-level conservatism was also associated with living in a state that had fewer laws protecting transgender rights or more antitransgender laws (*r* = −.81). These analyses reveal a consistent association between conservatism, both at the state and individual level, with living in a region that had more antitransgender policies. As a result, including conservatism as a predictor of anti-transgender legislation risks nullifying or reducing the strength of the association for our measures of anti-transgender prejudice.

Indeed, in analyses that included individual political ideology, the relationship between state policy tally and implicit, *b* = 0.0004, 95% CI [−0.00006, 0.0009], *t*(40.88) = 1.69, *R*^2^ = .07, *p* = .098, and explicit attitudes, *b* = −0.0009, 95% CI [−0.002, 0.0002], *t*(37.87) = −1.56, *R*^2^ = .04, *p* = .127, was attenuated. Similar attenuation was seen using state-level conservatism: implicit, *b* = −0.002, 95% CI [−0.003, −0.0005], *t*(43.1) = −2.68, *R*^2^ = .003, *p* = .010, and explicit, *b* = −0.003, 95% CI [−0.006, 0.0005], *t*(38.86) = −1.66, *R*^2^ = .01, *p* = .105, though the coefficient for implicit bias was statistically significant.

These data suggest that it is difficult to disentangle the causal relationships between overall state-level conservatism, individual-level conservatism of residents in that state, the extent to which a state adopts conservative legislative policy toward transgender people, and individual-level attitudes toward transgender people. Therefore, establishing causal models of whether state policy caused individual attitudes, or whether attitudes caused that state policy, is not possible with the cross-sectional and correlational nature of the present data and research design. And indeed, we suspect the relationship to be bidirectional to some extent. Future research adopting other designs can better estimate the causal relationship between these variables. We return to this issue in greater detail in the General Discussion.

## Discussion

Together, results suggest more discriminatory policies toward transgender people within a state were related to greater antitransgender implicit and explicit attitudes within individuals in those states. This work is the first to describe broad regional patterns of antitrans attitudes, and adds to the existing literature using regional outcomes concerning intergroup treatment as predictors of individual differences in intergroup biases (e.g., Hehman et al., 2018, 2019; Riddle & Sinclair, 2019; Stelter et al., 2022).

When looking at transgender attitudes specifically, this research adds to existing work concerning the relationship between policy beliefs and attitudes about gender identity. For instance, Axt et al. (2020) found that a relative preference for cisgender over transgender people in implicit and explicit attitudes was associated with higher levels of transphobia and weaker support for more inclusive policies concerning the treatment of transgender people. In this study, we extend this line of work by showing that states with more antitransgender policies also have individual residents who have greater antitransgender prejudices. Our use of a regional-level of analysis is especially pertinent because policies and laws that are passed impact entire areas (counties, states, etc.).

One of the most significant contributions of this research is that it speaks to the potential role of policy-making in changing attitudes and reducing prejudice. Generally, people infer that policies are prescriptive of the normative behavior encouraged or not in a given region (Tankard & Paluck, 2016). For instance, one prior study found that after a university issued a ban on outdoor smoking, students reported that smoking was less tolerated by others on campus (Procter-Scherdtel & Collins, 2013). Changes in perceived social norms are believed to create substantial behavioral change because people strive to make accurate social judgments and avoid social rejection (Cialdini & Goldstein, 2004). As a result, new social norms can be signaled via policy-making institutions like the administration boards or legislative bodies (Tankard & Paluck, 2016). For example, prior work found that participants perceived same-sex marriage as more socially acceptable following the U.S. Supreme Court ruling in favor of same-sex marriage legalization (Tankard & Paluck, 2017). Interestingly, this change in social norm perception happened independently of a change in personal attitudes (i.e., even people whose attitudes did not change following the court ruling still reported that societal norms had changed). This work has since been extended to show that implicit and explicit attitudes about gay people improved faster following the legalization of same-sex marriage (Ofosu et al., 2019). A similar pattern of results was found with explicit attitudes in Europe, following the passing of progressive same-sex relationship recognition policies (Aksoy et al., 2020).

Our findings on the relationship between transgender policy tally and transgender attitudes are in line with this prior work. However, while we draw from prior theoretical models that put forth a causal relationship between laws changing individual attitudes (e.g., Nilsson et al., 2016; Tankard & Paluck, 2017), it is important to demarcate the correlational nature of our data that limit our ability to make such a causal argument. Higher state-level negative transgender implicit or explicit bias could be either the cause, consequence, or both, of having zero to few policies protecting transgender rights. That is, while individuals may use their state’s treatment of transgender people as a factor in forming their own attitudes, it is also possible that states that have a greater number of residents with antitransgender attitudes choose to enact more discriminatory laws. We consider both explanations to illuminate the relationship between individual differences in prejudice and structural forms of discrimination.

The current analyses focused on a policy score that used the overall legislative environment (i.e., a sum value from several subdomains). It is possible that the observed results may have been most driven by transgender-related policies in a specific climate (e.g., in treatment of LGBT youth), though no theoretical accounts currently exist that would predict such a result. To investigate this issue, we ran analyses that separately used each of the seven subscores of the MAP Score (retrieved November 1, 2023): relationship and parental recognition, nondiscrimination, religious exemption laws, LGBT youth, health care, criminal justice, and identity documents. Results found that each subscore reliably predicted implicit and explicit attitudes when tested separately (see online supplement for full reporting).

These exploratory results illustrate the robustness of our effects, but further conclusions are complicated by the likely lack of independence among subscores; for instance, states passing antitransgender legislation in one domain could be expected to do so in other domains as well. Indeed, a correlation matrix among subscores found that each score was correlated at minimum *r* = .37, with a median correlation of *r* = .70. As a result, it is difficult to tell whether, for example, residents were impacted by each instance of legislation concerning transgender people or if certain laws (e.g., those targeting LGBT youth) were particularly powerful. Future research will benefit from studies that can better answer this question (e.g., longitudinal designs that track individual prejudices before and after the passing of specific laws).

While previous research has found similar patterns of results from only a single, highly salient law (e.g., Aksoy et al., 2020; Ofosu et al., 2019), these results are consistent with a model of perceived norms causing attitude change. Here, in the case of transgender policies, that effect may manifest as many laws or many categories of laws contributing to the perceived norm. In other cases, extremely salient single cases might shape perceived norms. Still, it is worth noting that these additional analyses were exploratory, and thus not motivated by theory. Given the large number of analyses run, we are also cautious about any conclusions, given the inflated risk of Type I error.

### The Role of Political Ideology

In a model including participant’s political ideology, we found that relationships in some models between transgender attitudes and related state policies became nonsignificant. To some, this may render the other results reported here unsurprising or uninteresting, as the association between individual attitudes and state-level policies can be “explained by” conservatism, both at the resident level and the state level. However, while we report results with conservatism as a covariate, conceptually it may make little sense to include the variable in our model, since we do not believe that political ideology is necessarily a *confounder* variable, meaning a variable that is causally related to both the dependent and independent variable (Wysocki et al., 2022). There is currently no causal evidence that conservatism in and of itself *causes* transgender biases, and research shows that conservatism is not uniformly associated with intergroup prejudice (Brandt, 2017). Thus, while political ideology is certainly associated with prejudice toward transgender people, we believe that the field currently lacks the causal rationale to view it as a confounding variable in our model.

Given the strong relationship with state-level political conservatism, one concern would be that people are reporting transgender attitudes that are consistent with the perceived attitudes of those around them due to social desirability. The convergence of both implicitly and explicitly measured forms of bias helps to allay this concern. While responses on a self-report scale are certainly under an individual’s conscious control, more automatic implicit associations are less subject to social desirability concerns (e.g., Nosek, 2005). Accordingly, we consider the convergence between explicit and implicit attitudes in the present research to buttress the argument that policy is related to individual biases.

Another possible concern is that regional conservatism, rather than legislation about the treatment of transgender people, is responsible for shifts in transgender attitudes. While this perspective would argue for a different cause in what is responsible for creating prejudice toward transgender people, it is still very much consistent with the broader idea of norms influencing attitudes. Importantly, recent papers have described how public views on transgender issues may be more influenced by polarized discourse from political elites than gay and lesbian issues have been (Haider-Markel et al., 2019; Jones & Brewer, 2020). Thus, norms may be conveyed by the policies that are passed, but can also be conveyed through other, related means (e.g., news coverage that depicts transgender people in a negative light). Indeed, our arguments about the relationship between norms and prejudice would hold if later studies found that shifts in regional conservatism (rather than the passing of antitransgender laws) were more predictive of changes in individual transgender attitudes, particularly if conservatism is increasingly associated with antitransgender policies and rhetoric.

### Future Directions

The scope of our research is constrained by the scope of our data. Future research in this area may want to focus on questions related to long-term change or stability. With the Transgender IAT being among the most recently developed implicit attitude measures on Project Implicit, it is yet unknown what trend these attitudes are following (i.e., whether antitransgender biases are increasing or decreasing). For instance, in a 14-year review of data collected on several IATs, Charlesworth and Banaji (2022) found that implicit attitudes have been moving toward neutrality in some domains (e.g., sexual orientation, race, and skin tone), while others have remained stable (e.g., age, disability, and body-weight). Regarding gender stereotypes, implicit and explicit attitudes have moved toward neutrality as much as 19% over the last decade (Charlesworth & Banaji, 2021). In light of this evidence, we could expect to see transgender attitudes moving toward neutrality in the coming years because people usually report similar attitudes with sexual minorities (Norton & Herek, 2013), though the rise of discriminatory laws concerning transgender people may signal an increase in antitransgender attitudes.

The findings of this analysis are also limited by the use of a single measure (the IAT) to assess implicit attitudes. In recent years, many have critiqued the IAT’s validity as an attitude measure, such as through citing its weak relationship with other supposedly related measures or with measures of intergroup behavior (Greenwald et al., 2022; Kurdi et al., 2019). In addition, the degree to which performance on the IAT reflects purely associative (versus propositional) information remains unclear (e.g., Mann et al., 2019; Rothermund & Wentura, 2004). Our conclusions would then be strengthened from future research that uses other indirect measures (e.g., Axt et al., 2023) or relies on different indicators of structural antitransgender prejudice (e.g., higher levels of suicidal thinking among transgender people in a given area; Price et al., 2024).

To this end, future studies could adopt a longitudinal or quasi-experimental approach to better understand how changes in policy impact transgender implicit attitudes. These studies would provide more causal evidence concerning the relationship between regional policies and individual prejudices (as well as the causal relationship between conservatism and transgender prejudices). For instance, future research on transgender implicit attitudes and policing could proceed similarly to Ofosu et al. (2019) and conduct a time series analysis based on state-by-state policy changes regarding the treatment of transgender people. Many state legislatures are currently evaluating important transgender-related policies, such as the banning of transgender youth from participating in sports according to their gender identity (AP News, 2020). Different states will likely remove discriminatory policies and adopt policies protecting transgender rights at different rates. Therefore, future studies could look into the pre- versus post-policy effect that such changes have on implicit transgender attitudes. Such analyses could better clarify the causal relationship between policy-making and transgender attitudes, though this study accomplishes the important first step of identifying a clear relationship between regional estimates of implicit transgender attitudes and the enactment of policies that combat or increase discrimination against transgender people.

Finally, future studies should expand on the impact of legislation on the lives of transgender individuals. Importantly, we would need to validate how the enactment of discriminatory laws affects those groups, if we wish to provide conclusive arguments against them. Indeed, one study has found that greater structural transphobia in a state, operationalized as an aggregate measure of various transphobic policies and attitudes at the state-level, was associated with greater psychological distress of its trans-identifying residents (Price et al., 2024). While much more research is needed on this question, these findings demonstrate that legislations can have a real and measurable impact on transgender people’s lives.

## Conclusion

This research provides evidence of a relationship between policy characteristics and implicit and explicit attitudes about transgender people. In states with less discriminatory laws, implicit and explicit attitudes toward transgender people were more favorable. These findings represent a critical step in understanding how biases against transgender persons relate to regional factors like laws, paving the way for future research looking at long-term patterns of change in transgender attitude and their relation with policy-making.

## Supplemental Material

sj-docx-1-psp-10.1177_01461672231218340 – Supplemental material for Local Legislation is Associated With Regional Transgender Attitudes Supplemental material, sj-docx-1-psp-10.1177_01461672231218340 for Local Legislation is Associated With Regional Transgender Attitudes by Eliane Roy, Eric Hehman and Jordan Axt in Personality and Social Psychology Bulletin

## Notes

1. We performed additional analyses to verify that primary results were not impacted by the temporal distance between when participants completed the Transgender IAT (between 2020 and 2022) and the July 2022 timepoint we used for the MAP scores. Specifically, we ran separate models that treated the number of months elapsed between when attitudes were measured and when the policy values were retrieved as a moderator. Months elapsed did not moderate any relationship between resident-level prejudice and state-level policies. See online supplement for full reporting of results.
