# Supplementary material for: Local Legislation is Associated With Regional Transgender Attitudes
Source: Pers Soc Psychol Bull. 2023 Dec 28;51(8):1361–73. doi: 10.1177/01461672231218340 (PMC12206243; doi:10.1177/01461672231218340)
Supplement: sj-docx-1-psp-10.1177_01461672231218340 – Supplemental material for Local Legislation is Associated With Regional Transgender Attitudes [file sj-docx-1-psp-10.1177_01461672231218340.docx]

Local Legislation is Associated with Regional Transgender Attitudes

Supplementary Materials

**Appendix A**: Predicting transgender implicit and explicit attitudes from transgender state policy tally for full and cisgender-only samples with either implicit or explicit attitudes as covariate. (p. 2-3)

**Appendix B**: Predicting transgender implicit and explicit attitudes from transgender state policy tally for full and cisgender-only samples, controlling for demographics. (p. 4-6)

**Appendix C**: Correlation matrix of state conservatism, conservatism of individuals, and anti-transgender laws. (p. 7)

**Appendix D**: Predicting transgender implicit and explicit attitudes from transgender state policy tally for full and cisgender-only samples, controlling for state-level conservatism and individual-level political ideology. (p. 8-9)

**Appendix E**: Predicting transgender implicit attitudes from transgender state policy tally and the months elapsed between taking the IAT and the policy score. (p. 10)

**Appendix F**: Predicting transgender explicit attitudes from transgender state policy tally and the months elapsed between taking the IAT and the policy score. (p. 11)

**Appendix G**: Predicting transgender implicit attitudes from different from the category specific transgender state policy tallies. (p. 12)

**Appendix H**: Predicting transgender explicit attitudes from different from the category specific transgender state policy tallies. (p. 13)

**Appendix A**

Predicting transgender implicit and explicit attitudes from transgender state policy tally for full and cisgender-only samples with either implicit or explicit attitudes as covariate.

We included these models in the supplemental materials to show how much variance can be explained by implicit or explicit attitudes when the other is included as a covariate. It is worth noting that we do not think these models are an ideal test of our hypothesis, given the high correlation (r = .35) between transgender implicit attitudes and the explicit aggregated attitude score. As such, we think that the following models do not make a clear theoretical contribution. In such models, we are predicting a measure of bias from another measure of bias, and then examining whether any leftover variance might be associated with regional variation in the anti-transgender legislation. Importantly though, the strong relationship between the two measures of bias is interfering with our ability to test the true association between the legislation and individual biases.

I

| **Table 1.** Predicting transgender explicit attitudes from transgender implicit attitudes and transgender state policy tally for full and cisgender-only samples. | | | | | |
| --- | --- | --- | --- | --- | --- |
| Parameter or Estimate |  | Explicit Attitudes | | | |
|  |  |  | Full Sample |  | Cis-only Sample |
|  |  |  |  |  |  |
| *Fixed effects* |  |  |  |  |  |
| Intercept |  |  | -.049 (.01)*** |  | .063 (.01)*** |
| Trans. Legislation |  |  | -.008 (.00)*** |  | -.007 (.00)*** |
| Implicit attitudes |  |  | .780 (.00)*** |  | .617 (.00)*** |
|  |  |  |  |  |  |
| *Variance components* |  |  |  |  |  |
| State intercept variance |  |  | .003 (.06) |  | .003 (.06) |
| Residual variance |  |  | .865 (.93) |  | .746 (.86) |
|  |  |  |  |  |  |
| Estimated Fixed R^2^ |  |  | .13 |  | .62 |
|  |  |  |  |  |  |
| *Note*. *** = *p* < .001. ** = *p* < .01 | | | | | |

| **Table 2.** Predicting transgender implicit attitudes from transgender explicit attitudes and transgender state policy tally for full and cisgender-only samples. | | | | | |
| --- | --- | --- | --- | --- | --- |
| Parameter or Estimate |  | Implicit Attitudes | | | |
|  |  |  | Full Sample |  | Cis-only Sample |
|  |  |  |  |  |  |
| *Fixed effects* |  |  |  |  |  |
| Intercept |  |  | .108 (.00)*** |  | .123 (.00)*** |
| Trans. Legislation |  |  | -.003 (.00) |  | -.0006 (.00) |
| Explicit attitudes |  |  | .157 (.00)*** |  | .143 (.00)*** |
|  |  |  |  |  |  |
| *Variance components* |  |  |  |  |  |
| State intercept variance |  |  | .0002 (.01) |  | .003 (.06) |
| Residual variance |  |  | .174 (.42) |  | .746 (.86) |
|  |  |  |  |  |  |
| Estimated Fixed R^2^ |  |  | .12 |  | .09 |
|  |  |  |  |  |  |
| *Note*. *** = *p* < .001. ** = *p* < .01 | | | | | |

**Appendix B**

Predicting transgender implicit and explicit attitudes from transgender state policy tally for full and cisgender-only samples, controlling for demographics.

| **Table 3.** Predicting transgender implicit attitudes from transgender state policy tally and selected demographic covariates for full samples. | | | | | | |
| --- | --- | --- | --- | --- | --- | --- |
|  |  |  |  |  |  |  |
| Parameter or Estimate |  | Models with Covariate | | | | |
|  |  | Gender |  | Race |  | Age |
|  |  |  |  |  |  |  |
| *Fixed effects* |  |  |  |  |  |  |
| Intercept |  | .12*** |  | .12*** |  | .12*** |
| Trans. Legislation |  | -.002*** |  | -.002*** |  | -.002*** |
| Gender |  | .006*** |  | **—** |  | **—** |
| Race |  | **—** |  | .01*** |  | **—** |
| Age |  | **—** |  | **—** |  | .007*** |
|  |  |  |  |  |  |  |
| *Variance components* |  |  |  |  |  |  |
| State intercept variance |  | .0003 (.02) |  | .0003 (.02) |  | .0004 (.02) |
| Residual variance |  | .20 (.45) |  | .20 (.45) |  | .19 (.44) |
|  |  |  |  |  |  |  |
|  |  |  |  |  |  |  |
| *Note*. Age was centered using grand-mean. Gender was coded as Female = -1 and Male = 1. Race was coded as White = -1 and non-White = 1. *** = *p* < .001. ** = *p* < .01 | | | | | | |

| **Table 4.** Predicting transgender explicit attitudes from transgender state policy tally and selected demographic covariates for full samples. | | | | | | |
| --- | --- | --- | --- | --- | --- | --- |
|  |  |  |  |  |  |  |
| Parameter or Estimate |  | Models with Covariate | | | | |
|  |  | Gender |  | Race |  | Age |
|  |  |  |  |  |  |  |
| *Fixed effects* |  |  |  |  |  |  |
| Intercept |  | .04*** |  | .04*** |  | .04*** |
| Trans. Legislation |  | -.009*** |  | -.009*** |  | -.01*** |
| Gender |  | -.02*** |  | **—** |  | **—** |
| Race |  | **—** |  | .0008 |  | **—** |
| Age |  | **—** |  | **—** |  | .006*** |
|  |  |  |  |  |  |  |
| *Variance components* |  |  |  |  |  |  |
| State intercept variance |  | .004 (.07) |  | .004 (.07) |  | .005 (.07) |
| Residual variance |  | .99 (1) |  | 1 (1) |  | .98 (.99) |
|  |  |  |  |  |  |  |
|  |  |  |  |  |  |  |
| *Note*. Age was centered using grand-mean. Gender was coded as Female = -1 and Male = 1. Race was coded as White = -1 and non-White = 1. *** = *p* < .001. ** = *p* < .01 | | | | | | |

| **Table 5.** Predicting transgender implicit and explicit attitudes from transgender state policy tally and all demographic covariates for full samples. | | | |
| --- | --- | --- | --- |
|  | Model results | | |
|  |  | Implicit attitudes | Explicit attitudes |
| *Fixed effects* |  |  |  |
| Intercept |  | .132*** | .046*** |
| Trans. Legislation |  | -.002*** | -.010*** |
| Gender |  | .014*** | -.011*** |
| Race |  | .022*** | .012*** |
| Age |  | .007*** | .007*** |
|  |  |  |  |
| *Variance components* |  |  |  |
| State intercept variance |  | .0004 (.02) | .0005 (.07) |
| Residual variance |  | .19 (.44) | .99 (.995) |
|  |  |  |  |
| *Notes*. Age was centered using grand-mean. Gender was coded as Female = -1 and Male = 1. Race was coded as White = -1 and non-White = 1. *** = *p* < .001. ** = *p* < .01 | | | |

**Appendix C**

Correlation matrix of state conservatism, conservatism of individuals, and anti-transgender laws.

| **Table 6.** Correlation matrix of state conservatism, conservatism of individuals, and anti-transgender laws. | | | | |
| --- | --- | --- | --- | --- |
| Variable | *M* | *SD* | 1 | 2 |
|  |  |  |  |  |
| 1. State-level Conservatism | .46 | .09 |  |  |
| 2. Individual-level Conservatism | 2.94 | 1.7 | .86 |  |
| 3. Transgender legislation | 10.63 | 9.28 | -.87 | -.81 |
|  |  |  |  |  |
| *Note*. *M* and *SD* are used to represent mean and standard deviation, respectively. | | | | |

**Appendix D**

Predicting transgender implicit and explicit attitudes from transgender state policy tally for full and cisgender-only samples, controlling for state-level conservatism and individual-level political ideology.

| **Table 7.** Predicting transgender implicit attitudes from transgender state policy tally and different political covariates for full samples. | | | | |
| --- | --- | --- | --- | --- |
|  |  |  |  |  |
| Parameter or Estimate |  | Political covariates | | |
|  |  | Individual-level political ideology |  | State-level consevatism |
|  |  |  |  |  |
| *Fixed effects* |  |  |  |  |
| Intercept |  | .11*** |  | .12*** |
| Trans. Legislation |  | -.0004 |  | -.002*** |
| Individual-level political ideology |  | .07*** |  | **—** |
| State-level conservatism |  | **—** |  | .005 |
|  |  |  |  |  |
| *Variance components* |  |  |  |  |
| State intercept variance |  | .0002 (.01) |  | .0003 (.02) |
| Residual variance |  | .18 (.43) |  | .20 (.45) |
|  |  |  |  |  |
|  |  |  |  |  |
| *Note*. Individual-level political ideology and state-level conservatism were grand-mean centered. *** = *p* < .001. ** = *p* < .01 | | | | |

| **Table 8.** Predicting transgender explicit attitudes from transgender state policy tally and different political covariates for full samples. | | | | |
| --- | --- | --- | --- | --- |
|  |  |  |  |  |
| Parameter or Estimate |  | Political covariates | | |
|  |  | Individual-level political ideology |  | State-level consevatism |
|  |  |  |  |  |
| *Fixed effects* |  |  |  |  |
| Intercept |  | .01* |  | .01 |
| Trans. Legislation |  | -.0009 |  | -.003 |
| Individual-level political ideology |  | .27*** |  | **—** |
| State-level conservatism |  | **—** |  | .66*** |
|  |  |  |  |  |
| *Variance components* |  |  |  |  |
| State intercept variance |  | .0009 (.03) |  | .003 (.06) |
| Residual variance |  | .78 (.89) |  | .99 (1) |
|  |  |  |  |  |
|  |  |  |  |  |
| *Note*. Individual-level political ideology and state-level conservatism were grand-mean centered. *** = *p* < .001. ** = *p* < .01. * = *p* < .05. | | | | |

**Appendix E**

Predicting transgender implicit attitudes from transgender state policy tally and the months elapsed between taking the IAT and the policy score.

| **Table 9.** Predicting transgender implicit attitudes from transgender state policy tally and the months elapsed between taking the IAT and the policy score. | |
| --- | --- |
| Parameter or Estimate | Implicit attitudes |
|  |  |
| *Fixed effects* |  |
| Intercept | .130*** |
| Trans. Legislation | -.002*** |
| Months elapsed | -.001*** |
| Trans. Legislation * Months elapsed | -. 000007 |
|  |  |
| *Variance components* |  |
| State intercept variance | .0003 |
| Residual variance | .199 |
|  |  |
| *Note*. *** = *p* < .001. | |

**Appendix F**

Predicting transgender explicit attitudes from transgender state policy tally and the months elapsed between taking the IAT and the policy score.

| **Table 10.** Predicting transgender explicit attitudes from transgender state policy tally and the months elapsed between taking the IAT and the policy score. | |
| --- | --- |
| Parameter or Estimate | Implicit attitudes |
|  |  |
| *Fixed effects* |  |
| Intercept | .059*** |
| Trans. Legislation | -.009*** |
| Months elapsed | -.001*** |
| Trans. Legislation * Months elapsed | .00003 |
|  |  |
| *Variance components* |  |
| State intercept variance | .004 |
| Residual variance | .992 |
|  |  |
| *Note*. *** = *p* < .001. | |

**Appendix G**

Predicting transgender implicit attitudes from the category specific transgender state policy tallies.

| **Table 11.** Predicting transgender implicit attitudes from the category specific transgender state policy tallies. | | | | | | | | | |
| --- | --- | --- | --- | --- | --- | --- | --- | --- | --- |
|  |  |  |  |  |  |  |  |  |  |
| Parameter or Estimate |  | 1 | 2 | 3 | 4 | 5 | 6 | 7 |  |
|  |  |  |  |  |  |  |  |  |  |
| *Fixed effects* |  |  |  |  |  |  |  |  |  |
| Intercept |  | .113*** | .114*** | .113*** | .114*** | .114*** | .114*** | .114*** |  |
| 1. Relationship and parental recognition |  | -.020** | — | — | — | — | — | — |  |
| 2. Non-discrimination |  | — | -.006*** | — | — | — | — | — |  |
| 3. Religious exemption laws |  | — | — | -.018*** | — | — | — | — |  |
| 4. LGBT youth |  | — | — | — | -.005*** | — | — | — |  |
| 5. Healthcare |  | — | — | — | — | -.005*** | — | — |  |
| 6. Criminal justice |  | — | — | — | — | — | -.009*** | — |  |
| 7. Identity documents |  | — | — | — | — | — | — | -.006*** |  |
|  |  |  |  |  |  |  |  |  |  |
| *Variance components* |  |  |  |  |  |  |  |  |  |
| State variance |  | .0004 | .0004 | .0003 | .0003 | .0004 | .0004 | .0004 |  |
| Residual variance |  | .199 | .199 | .199 | .199 | .199 | .199 | .199 |  |
|  |  |  |  |  |  |  |  |  |  |
| *Note*. All fixed predictors were grand-mean centered. *** = *p* < .001. *** = p < .01. | | | | | | | | | |

**Appendix H**

Predicting transgender explicit attitudes from the category specific transgender state policy tallies.

| **Table 12.** Predicting transgender explicit attitudes from the category specific transgender state policy tallies. | | | | | | | | |
| --- | --- | --- | --- | --- | --- | --- | --- | --- |
|  |  |  |  |  |  |  |  |  |
| Parameter or Estimate |  | 1 | 2 | 3 | 4 | 5 | 6 | 7 |
|  |  |  |  |  |  |  |  |  |
| *Fixed effects* |  |  |  |  |  |  |  |  |
| Intercept |  | .043** | .043*** | .043*** | .043*** | .042*** | .043*** | .042*** |
| 1. Relationship and parental recognition |  | -.102*** | — | — | — | — | — | — |
| 2. Non-discrimination |  | — | -.039*** | — | — | — | — | — |
| 3. Religious exemption laws |  | — | — | -.072*** | — | — | — | — |
| 4. LGBT youth |  | — | — | — | -.028*** | — | — | — |
| 5. Healthcare |  | — | — | — | — | -.028*** | — | — |
| 6. Criminal justice |  | — | — | — | — | — | -.054*** | — |
| 7. Identity documents |  | — | — | — | — | — | — | -.034*** |
|  |  |  |  |  |  |  |  |  |
| *Variance components* |  |  |  |  |  |  |  |  |
| State variance |  | .009 | .006 | .009 | .006 | .005 | .006 | .006 |
| Residual variance |  | .992 | .992 | .992 | .992 | .992 | .992 | .992 |
|  |  |  |  |  |  |  |  |  |
| *Note*. All fixed predictors were grand-mean centered. *** = *p* < .001. *** = p < .01. | | | | | | | | |
